# X-ray Thermo-Diffraction Study of the Aluminum-Based Multicomponent Alloy Al_58_Zn_28_Si_8_Mg_6_

**DOI:** 10.3390/ma15145056

**Published:** 2022-07-20

**Authors:** Yoana Bilbao, Juan José Trujillo, Iban Vicario, Gurutze Arruebarrena, Iñaki Hurtado, Teresa Guraya

**Affiliations:** 1 Department of Mining and Metallurgical Engineering and Materials Science, Faculty of Engineering of Bilbao, University of the Basque Country (UPV/EHU), 48013 Bilbao, Spain; teresa.guraya@ehu.eus; 2 Mechanical and Manufacturing Department, Faculty of Engineering, Mondragon Unibertsitatea, 20500 Arrasate/Mondragon, Spain; jjtrujillo@mondragon.edu (J.J.T.); garruebarrena@mondragon.edu (G.A.); ihurtado@mondragon.edu (I.H.); 3 Manufacturing Processes and Materials Department, Tecnalia, Basque Research and Technology Alliance (BRTA), 48160 Derio, Spain; iban.vicario@tecnalia.com

**Keywords:** lightweight multicomponent alloys, X-ray thermo-diffraction, differential scanning calorimetry, Al–Zn, Zn precipitation, Mg–Zn phases, strontium modification

## Abstract

Newly designed multicomponent light alloys are giving rise to non-conventional microstructures that need to be thoroughly studied before determining their potential applications. In this study, the novel Al_58_Zn_28_Si_8_Mg_6_ alloy, previously studied with CALPHAD methods, was cast and heat-treated under several conditions. An analysis of the phase evolution was carried out with in situ X-ray diffraction supported by differential scanning calorimetry and electron microscopy. A total of eight phases were identified in the alloy in the temperature range from 30 to 380 °C: α-Al, α’-Al, Zn, Si, Mg_2_Si, MgZn_2_, Mg_2_Zn_11_, and SrZn_13_. Several thermal transitions below 360 °C were determined, and the natural precipitation of the Zn phase was confirmed after nine months. The study showed that the thermal history can strongly affect the presence of the MgZn_2_ and Mg_2_Zn_11_ phases. The combination of X-ray thermo-diffraction with CALPHAD methods, differential scanning calorimetry, and electron microscopy offered us a satisfactory understanding of the alloy behavior at different temperatures.

## 1. Introduction

Historically, metallic alloys have been developed by selecting one or two major components and adding several minor ones that confer specific properties, such as corrosion resistance or higher mechanical properties. The multicomponent alloy concept, however, is based on the design of alloys where there are several main components that cover the central areas of phase diagrams [1].

Initial developments in the field focused mainly on steel-like alloys for industrial applications. They were based on equiatomic and near-equiatomic compositions of Co, Cr, Cu, Fe, Mn, or Ni, sometimes adding Al, Ti, or Zr, that resulted in single or dual phase microstructures [2,3,4,5,6,7]. Yeh et al. suggested that the prevalence of solid solutions over intermetallic phases could be explained by the high mixing entropy generated by the multiple components in the alloy, hence the term “high-entropy alloys” (HEAs) [8]. The conditions that the alloys should satisfy to be considered HEAs may be found in [9].

Over the past decade, research has been extended to other alloy classifications that have evolved from the original HEA concept: “medium-entropy alloys” (MEAs) [9], “non-equiatomic HEAs”, or “multi-phase HEAs” that may contain bulky secondary phases [10]. In fact, the idea of intentionally having secondary phases in this type of alloy was first suggested by Miracle et al. [11]. Other alloy families have also been explored, leading to refractory metal HEAs for high temperature structural applications based on Cr, Hf, Mo, Nb, Ta, Ti, V, and W or lightweight multicomponent alloys in the aeronautical field involving Al, Li, Mg, or Zn. Several studies may be found in the literature as proof of this tendency [12,13,14,15,16,17]. 

Among the lightweight multicomponent alloys, Yang et al. explored the Al–Li–Mg–(Zn, Cu, Sn) system, obtaining structures dominated by intermetallic compounds. The aluminum face-centered cubic (FCC) structure predominated only in selected alloy compositions [18]. In fact, Sanchez et al. highlighted the difficulty of forming solid solutions in medium entropy alloys based on aluminum (65–70 at. %) with elements such as Cu, Mg, Cr, Fe, Si, Ni, Zn, or Zr. The magnitude of the negative mixing enthalpy of aluminum with transition metals gave rise to intermetallic phases [19]. The presence of intermetallics was also reported by Tun et al. [20]. Only the most recent research suggests that rapid solidification processes may enhance single phase microstructures in this type of alloy [21]. However, in a previous work by Nagase et al. on Al–Mg–Li–Ca equiatomic and non-equiatomic alloys, a single solid solution could not be obtained, even with rapid solidification [22].

Asadikiya et al. considered that the application of the entropy concept in aluminum alloys may be the answer to the challenge of developing novel Al alloys with improved properties [10]. Therefore, multicomponent lightweight alloys continue to be researched for their potential applications.

In the present study, the objective was to characterize the novel Al_58_Zn_28_Si_8_Mg_6_ cast alloy. It was designed to obtain as much solid solution of aluminum and zinc as possible, reinforced with intermetallics based on Zn, Mg, and Si. On the one hand, zinc is highly soluble in aluminum, enhancing the obtention of a solid solution matrix. On the other hand, Mg–Zn phases are the usual precipitates in 7xx.x aluminum cast alloys, while Mg–Si phases are common in 3xx.x alloys. In addition, Al–Zn-based alloys have attracted the interest of researchers beyond their usual use as coatings. In fact, Al–Zn cast alloys have potential applications where tribological and damping properties are required [23,24,25]. In terms of entropy, our multicomponent alloy would be classified as a multi-phase MEA. 

The approach was explored by the CALPHAD (calculation of phase diagrams) method. This technique requires databases that are valid in composition ranges that may not be found in conventional alloys, thus demanding further experimental verification [26]. However, it is considered the most direct method for compositional design [27] and has already been used in the design of lightweight multicomponent alloys with differing degrees of success [28,29,30].

The study is focused on identifying and evaluating the effect of the temperature on the phases that are generated at different initial thermal conditions. Differential scanning calorimetry (DSC), electron microscopy, and X-ray thermo-diffraction are the techniques used in this evaluation. X-ray thermo-diffraction, also known as “high temperature X-ray diffraction” (HT-XRD), enables the in situ study of the solution and precipitation phenomena in the alloys [31,32,33].

## 2. Materials and Methods

### 2.1. Material Manufacturing

Aluminum was melted at 750 °C in a resistance furnace with forced convection; silicon and zinc were subsequently added. Magnesium followed, and once all the elements were melted, strontium was added as a silicon modifier. Aluminum, magnesium, and silicon were of commercial purity, whereas zinc was incorporated by adding a Zamak Zn4Al1Cu alloy so that the final alloy composition contained some residual copper. Samples were obtained to determine the chemical composition by inductively coupled plasma mass spectrometry (ICP) (Table 1). 

The metal was gravity cast into a graphite mold, and samples were obtained that were 50 mm long, 22.5 mm wide, and 4 mm thick.

### 2.2. Selection of Sample Thermal Treatments and Study Temperatures

**Figure 1:**
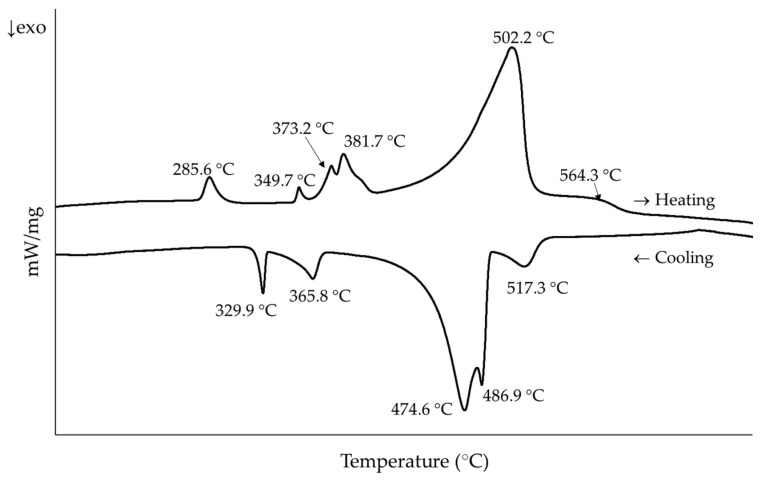
DSC curves for the as-cast sample. Peak temperatures during heating were considered for the thermal treatment selection of the samples.

In order to select the temperatures of interest, DSC tests were performed on as-cast samples (Figure 1). A Netzsch STA 449 Fe Jupiter calorimeter was used, and measurements were made under argon atmosphere in a temperature range between 25 and 675 °C with a heating rate of 10 °C/min. Samples were then cooled down back to room temperature under these same conditions. 

**Figure 2:**
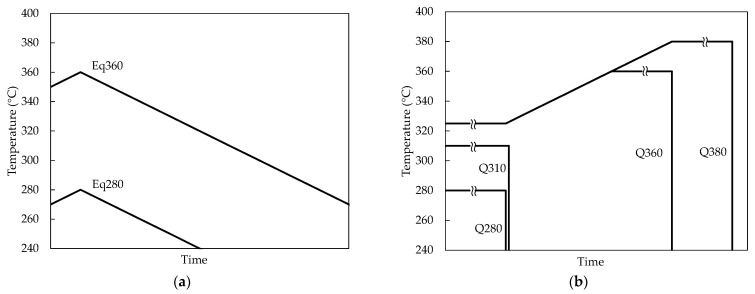
Heating and cooling sequences for the samples, in addition to the as-cast sample. (**a**) Slowly cooled (10 °C/min) samples (Eq280 and Eq360). (**b**) Quenched samples (Q280, Q310, Q360, and Q380).

We decided to subject the samples to seven different thermal conditions to try to separate and simplify the identification of the different phases appearing and disappearing during the heating process (Table 2 and Figure 2).

### 2.3. Thermodynamic Simulations

Equilibrium and Scheil non-equilibrium solidification simulations were carried out with the CALPHAD method for the cast alloy composition with FactSage 7.3 software, along with the FTlite (2021) database. Only the four main elements in the alloy were considered.

### 2.4. Microstructural Observations

As-cast and Q380 samples were observed with scanning electron microscopy (SEM of Shottky field emission, JEOL JSM-7000F) and energy dispersive X-ray spectroscopy (INCA EDX detector X-sight Serie Si (Li) pentaFET Oxford) at an electron beam voltage of 5.0 kV at room temperature. Specimens had been previously cleaned, ground, and polished to obtain a proper surface finish for the analysis.

### 2.5. X-ray Thermo-Diffraction Tests

The equipment used for the X-ray thermo-diffraction tests was a Bruker D8 Advance diffractometer that operated at 30 kV and 20 mA for reflection measurements. It was equipped with a copper anode (λ = 1.5418 Å), a Vantec-1 PSD detector, and an Anton Parr HTK2000 high temperature furnace. The sample holder used, on which the test temperature was controlled, was made of platinum.

**Figure 3:**
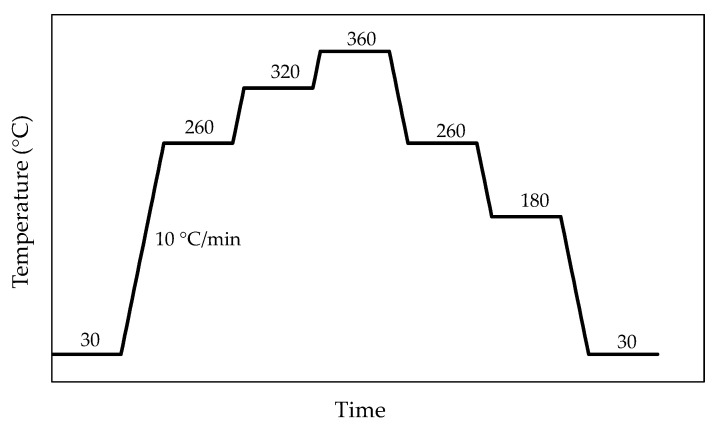
Thermal cycle of the samples in the thermo-diffractometer. Measurements were performed at the temperatures indicated in each step.

The seven specimens, which were 10 × 10 mm^2^ with a thickness between 1 and 2 mm, were subjected to a heating cycle from 30 to 360 °C and cooling again to 30 °C in the diffractometer. Diffraction tests were performed at room temperature (30 °C), at three temperatures during heating (260, 320, 360 °C), and at three temperatures during cooling (260, 180, 30 °C), based on the temperatures of interest found in the DSC curves (Figure 3). The measurements were recorded in the range 10° ≤ 2θ ≤ 100° at increments of 0.033°, with each stage lasting 0.8 s. 

Thermo-diffraction tests were performed three months after the samples were prepared. Twelve months after the preparation; that is, nine months after being subjected to the thermal cycle in the thermo-diffractometer, samples were retested in the same conditions as before but only at 30 °C, in order to observe whether natural precipitation had taken place.

The X-ray diffraction patterns were indexed with the PDF-4+ 2021 database from the International Center for Diffraction Data (ICDD). For the search of non-indexed phases, least squares-based Rietveld refinement was carried out in selected patterns with the FullProf software (FullProf.2k Version 7.40, January 2021, J. Rodriguez-Carvajal, ILL, Grenoble, France). The shape of the Bragg peaks was represented by a Pseudo-Voigt function. Conventional R-values, corrected for background, are given in the figures as agreement of the fitting to the observed values [34,35]. The term “intensity” is used to refer to the “integrated intensity”.

## 3. Results

### 3.1. Thermodynamic Simulation Results

**Figure 4:**
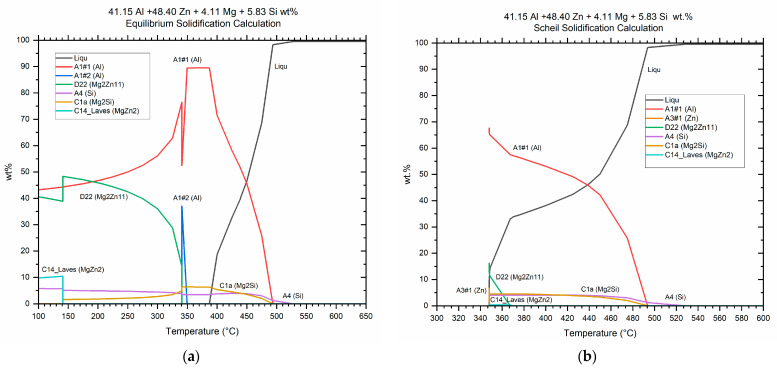
Thermodynamic simulations with FactSage for the studied alloy considering (**a**) equilibrium solidification conditions and (**b**) non-equilibrium solidification (Scheil model).

Thermodynamic simulations performed with FactSage for equilibrium cooling conditions (Figure 4a) predicted a high proportion of the FCC aluminum solid solution at temperatures between 360 and 380 °C, with the Si and Mg_2_Si phases being precipitated at these temperatures. As cooling went on, the solid solution decomposed and around 350 °C a second aluminum phase (Al#2) was generated but disappeared soon after. This phase would correspond to the zinc-rich α’ aluminum phase of the miscibility gap in the Al–Zn system [36,37]. At about 340 °C, the intermetallic phase Mg_2_Zn_11_ was formed, and MgZn_2_ precipitated from Mg_2_Zn_11_ at around 140 °C. The simulation under non-equilibrium conditions (Scheil approximation) predicted the precipitation of Mg_2_Zn_11_ and MgZn_2_ at about 370 °C and that of hexagonal zinc at 350 °C (Figure 4b).

### 3.2. Microstructure of the Samples Depending on the Initial Thermal Condition

**Figure 5:**
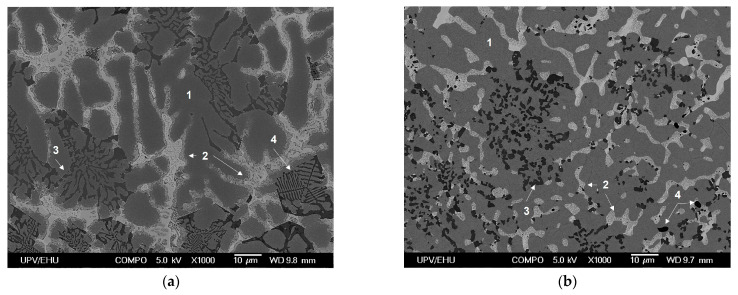

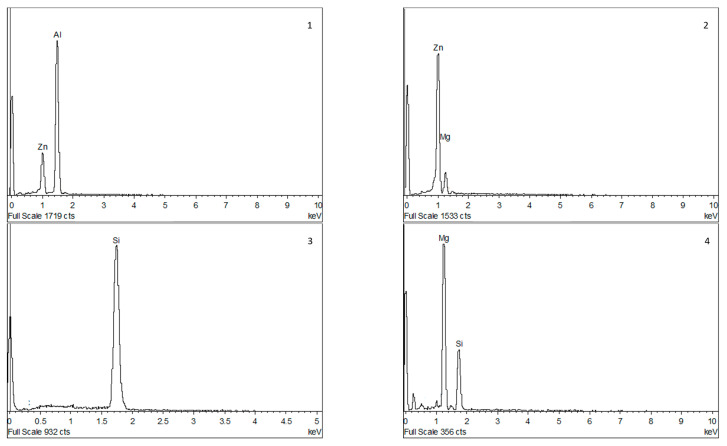
SEM micrographs of the material with ×1000 magnification (**a**) As-cast. (**b**) Q380. Numbers 1 to 4 refer to the EDX results provided below. 1: Al-Zn matrix, 2: Mg-Zn phases, 3: Si phases and 4: Mg-Si phases.

The microstructure resulting from the as-cast state was heterogeneous, with different phases distributed throughout the interdendritic region depending on the solidification rate (Figure 5a). In the Q380 condition (Figure 5b), the globulization and reduction in the size of the phases after the solution treatment were remarkable. The Si, Mg–Si, and Mg–Zn phases were found by EDX measurements. The Si phase solidified in certain areas as eutectic and in other areas as primary silicon. In addition, isolated Al–Fe–Mg–Si phases were detected.

**Figure 6:**
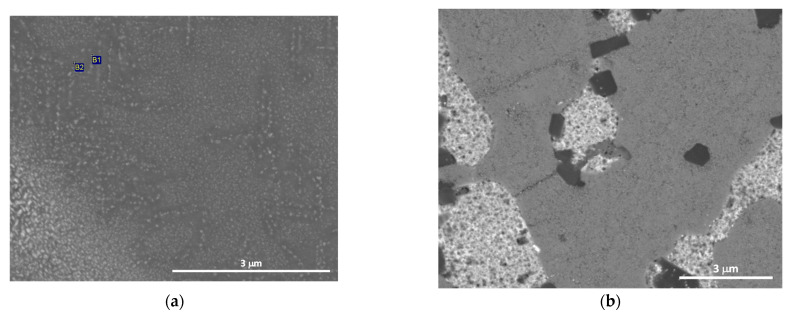
SEM micrographs of the Al–Zn matrix. (**a**) As-cast. (**b**) Q380.

As for the matrix, it showed a two-phase microstructure of aluminum and zinc. Precipitation of the Zn phase was observed in the as-cast material, unlike in sample Q380, where the Zn phase was not detected, indicating that it was dissolved within the matrix (Figure 6). 

**Figure 7:**
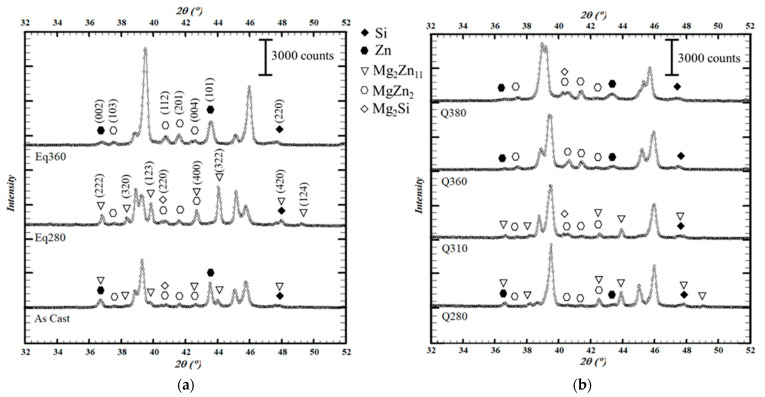
Diffraction patterns of the samples in each thermal condition at 30 °C prior to the heating cycle in the thermo-diffractometer. Indexation is shown above the patterns of samples Eq280 (Mg_2_Zn_11_ and Mg_2_Si phases) and Eq360 (Si, Zn and MgZn_2_ phases). Peaks corresponding to Al and Pt phases (the latter from the sample holder) are omitted for clarity. (**a**) As-cast samples and those cooled slowly. (**b**) Quenched samples.

The identification of the phases present in each initial thermal condition was performed by room temperature X-ray diffraction (before the heating cycle). As is shown in Figure 7, in addition to the Al phase (PDF: 00-004-0787) and the Pt phase from the sample holder (PDF: 04-013-4766), which are not indicated for clarity, the phases detected were Zn (PDF: 01-078-9363), Si (PDF: 00-027-1402), MgZn_2_ (PDF: 04-003-2083), Mg_2_Zn_11_ (PDF: 04-007-1412), and Mg_2_Si (PDF: 01-083-5235). 

However, the microstructure obtained depended on the applied treatment; that is, the temperature at which cooling had started and the cooling rate. In as-cast conditions Zn precipitated, as did both MgZn_2_ and Mg_2_Zn_11_ to a lesser extent. When slowly cooling from 280 °C (Eq280 sample), MgZn_2_ was obtained again, as in the previous case, but now Mg_2_Zn_11_ precipitated preferentially, while HCP Zn was hardly detected. When the cooling began at 360 °C (Eq360 sample), on the other hand, no precipitation of Mg_2_Zn_11_ was observed and zinc was present in the HCP Zn and MgZn_2_ phases. MgZn_2_ phases were found in greater quantities than in the as-cast or Eq280 conditions. As for the quenched samples, the Mg_2_Zn_11_ phase was dissolved when reaching 360 °C. 

Regarding the Fe-bearing quaternary phases observed by SEM, it was not possible to confirm them by X-ray diffraction. The most intense Bragg peak for Al_8_FeMg_3_Si_6_ (PDF: 03-065-5936) would overlap with the Al (111) reflection. Given its condition as a minor phase, further peaks could not be detected. Therefore, if other Cu- and Fe-bearing phases found in aluminum alloys containing Zn, Mg, Si, and/or Cu [38,39] were present in very small amounts in this alloy, specific X-ray diffraction conditions and equipment would be required to identify them. 

The appearance of the Mg_2_Si phase and the dissolution and precipitation of the Zn phase are discussed in the following section. 

### 3.3. Evolution of the HCP Zn and Intermetallic Phases with Temperature

**Figure 8:**
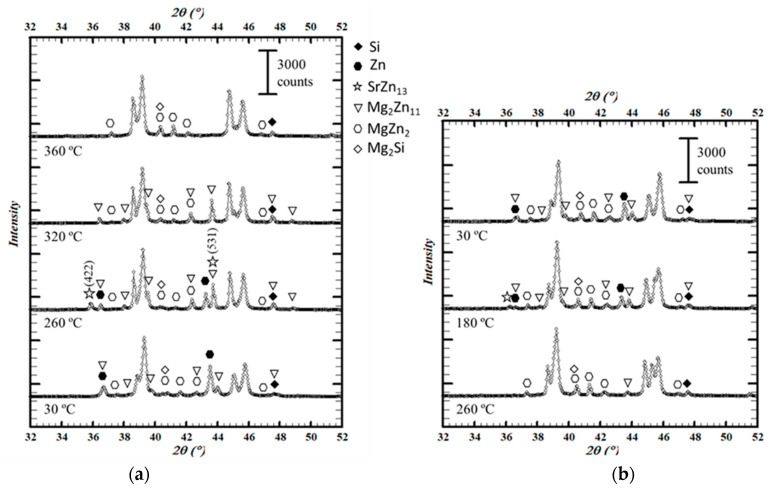
Diffraction patterns of the as-cast sample showing the evolution of the intermetallic phases, Zn, and Si with temperature (**a**) during the heating cycle (from 30 to 360 °C) (**b**) and cooling cycle (from 360 to 30 °C) in the thermo-diffractometer. The main Bragg peaks for the SrZn_13_ phase are identified.

The profiles obtained for the as-cast sample are representative of the evolution of the zinc-containing phases with temperature (Figure 8). The description is thus valid for the rest of the samples, while the matrix will be dealt with in the next section. This evolution is summarized below.

At 30 °C, Zn, MgZn_2_, and Mg_2_Zn_11_ phases were found. At 260 °C, the intensity of Zn peaks decreased while two additional Bragg peaks were detected around 2θ = 35.9° and 2θ = 54.0°. These peaks did not belong to any of the phases already indexed. Assuming they belonged to a new phase, it was clear that it arose at a temperature between 30 and 260 °C and likely dissolved between 260 and 280 °C, since it was absent in the Q280 sample at room temperature and in all the samples at any other temperature during the heating cycle. Indexing was performed considering minor elements present in the alloy, such as Cu, Fe, and Sr, and finally the SrZn_13_ phase was identified (PDF: 04-013-4885).

At 320 °C, both Zn and SrZn_13_ were dissolved. In addition, between 30 and 320 °C the intensity of Mg_2_Zn_11_ increased and then became negligible at 360 °C. From the increase in the intensity of the MgZn_2_ peaks at this temperature, it followed that Mg_2_Zn_11_ had not completely dissolved in the matrix and may have become the MgZn_2_ phase.

Regarding the cooling cycle, the onset of the precipitation of the Mg_2_Zn_11_ phase was observed at 260 °C, while that of the Zn phase was not detected until 180 °C. At this temperature, the peaks belonging to SrZn_13_ showed slightly and disappeared again with further cooling. It should be noted that in the final measurement at 30 °C, the distribution of precipitated phases was different from what it had been at the beginning. The proportion of MgZn_2_ obtained at 360 °C remained stable during cooling and was higher than that found during the initial measurement.

No evolution with temperature was observed for the Mg_2_Si phase. There were difficulties with detecting it in some of the measurements (see differences in Figure 7), but this was related to the specific sample (local segregations or inhomogeneities) and not to transformations taking place with temperature.

### 3.4. Evolution of Aluminum Phases

**Figure 9:**
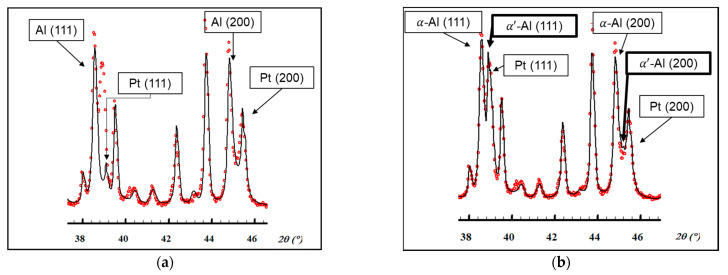
Rietveld fitting of the Eq280 sample at 30 °C (before heating) with FullProf software. Red dots: experimental data. Black line: Fitting data. (**a**) The following six phases are considered Al (a = 4.0428 Å), Zn, Si, MgZn_2_, Mg_2_Zn_11_, and Mg_2_Si. The Pt phase comes from the sample holder. R-values with background correction: R_p_ = 36.6 %, R_wp_ = 38.6 %, R_exp_ = 10.72 %, χ^2^ = 12.91. (**b**) An α’ phase with a lattice parameter a = 4.0089 Å is added to the previous case. Al phase in (a) is now labeled as α-Al. R-values with background correction: R_p_ = 20.5 %, R_wp_ = 18.7 %, R_exp_ = 10.54 %, χ^2^ = 3.15.

As was previously mentioned, a two-phase Al–Zn matrix was found. However, a detailed observation of the indexed profiles led to the detection of some peaks whose intensity was higher than expected. These observations were confirmed when performing a Rietveld fitting on one of the profiles (Eq280 sample at 30 °C, before heating). It was verified that some of the peaks could not be fitted with the original model and there was a phase missing (Figure 9a). The addition of a phase with the same spatial group as aluminum ($Fm\bar{3}m$) but a smaller lattice parameter managed to solve the structural model with satisfactory precision (Figure 9b). Due to the smaller atomic size of zinc compared to aluminum, a zinc-rich aluminum phase would show a smaller lattice parameter than α-Al and thus its Bragg peaks would shift to greater angles [32]. Therefore, the new phase observed could be the zinc-rich α’ aluminum metastable phase of the miscibility gap in the Al–Zn system. The samples were retested with room temperature X-ray diffractometry nine months later with the aim of determining whether this was the case, and it was found that precipitation of the Zn phase from the α’ metastable phase had taken place.

**Figure 10:**
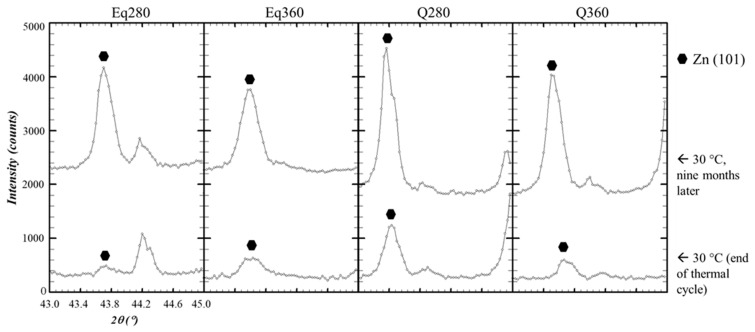
Close-up of the diffraction patterns of samples Eq280, Eq360, Q280, and Q360 showing the Bragg peaks of the (101) plane of the Zn phase. At the bottom, we show the profiles of the last measurements in the thermo-diffractometer at 30 °C at the end of the cooling cycle. At the top, we show the profiles obtained at the same temperature nine months later.

The evolution of the intensity of the Bragg peak corresponding to the (101) plane of the Zn phase was observed (Figure 10). The reason for choosing this peak is simple: it is the one with the maximum intensity of the Zn phase and it does not overlap with signals belonging to any other phase. For these reasons, this reflection is one of those taken as a reference in precipitation studies of Al–Zn alloys [31]. Intensity increased in all cases, although only four of them are shown in the figure.

## 4. Discussion

### 4.1. Phase Evolution with Temperature

The first transition temperature found in the DSC measurements (Figure 1) was 285 °C, and when compared with the evolution of the phases with the temperature observed in the diffraction patterns (Figure 8) it represents the dissolution of the Zn phase. Tests performed on Al–Zn samples with 24% atomic Zn estimate this reaction at 282 °C [32]. On the other hand, in the Al–Mg–Zn system the reaction would occur at 277 °C together with the partial dissolution of the Mg_2_Zn_11_ phase in the matrix [40]. However, it was not possible to determine to what extent this partial dissolution also takes place in the experimental samples at this temperature range.

The second temperature of interest in the DSC curve was about 350 °C. According to the analysis carried out, it corresponded to the complete dissolution of the Mg_2_Zn_11_ phase. This agrees with the precipitation temperature range expected for this phase by the equilibrium solidification simulations (Figure 4). In addition, as discussed in the analysis, at 360 °C a higher proportion of MgZn_2_ was observed so that it is possible that the dissolution of Mg_2_Zn_11_ enriched this phase. In fact, the reactions observed in the Al–Mg–Zn system fit this hypothesis, albeit at a lower temperature [40]. The reactions and their experimental and theoretical temperatures are collected in Table 3. 

**Figure 11:**
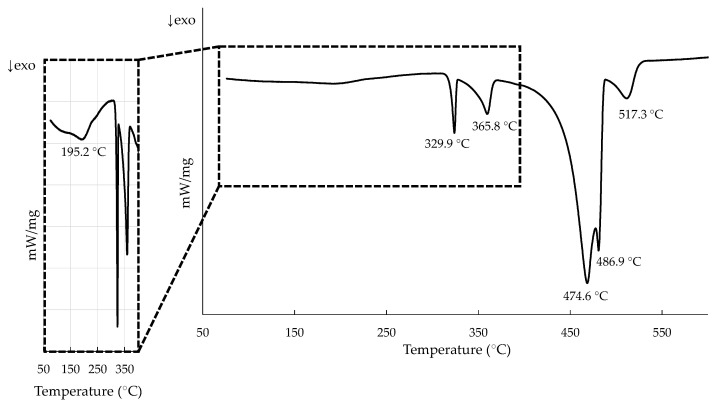
DSC curve for the as-cast sample during cooling. Compressed graph to highlight possible peak showing Zn phase precipitation.

During the cooling cycle in the diffractometer, zinc precipitation was observed at temperatures below 260 °C. However, such a transition was not easily detected in the DSC. According to the studies conducted by Skoko et al. with Al–Zn alloys [32], the precipitation transition occurs over a much larger temperature range than the dissolution one, so that the peak generated when cooling is much smaller. When looking specifically for this peak, it could be the one observed around 195 °C, a temperature consistent with the observations in the diffraction tests (Figure 11).

It should be noted that thermodynamic simulations did not agree with the experimental results regarding the MgZn_2_ and Zn phases.

### 4.2. Aluminum Phases and Ageing

According to the equilibrium solidification simulations for the alloy under study, phase Al#2, considered the zinc-rich aluminum α’ phase, should have arisen around 350 °C and decomposed shortly thereafter. The simulation with Scheil’s approach did not even foresee its appearance. Therefore, the observation of this phase at room temperature was not expected.

However, an additional phase was observed at 30 °C in all samples subjected to the different thermal conditions (Figure 9). After the heating and cooling cycles in the diffractometer, it was still detected. Since this phase would have the same crystal structure as the α-Al phase but a smaller lattice parameter, our first hypothesis was that it was the α’-Al phase.

Analyses carried out nine months later showed that the microstructure of the samples after the diffractometer cycle was metastable, with the Zn phase precipitating during this time (Figure 10). This fact confirmed that the precipitation process usually observed in Al–Zn alloys [31,32,37,41] had occurred, a phenomenon in which the α’ phase intervenes and that has been studied in detail with X-ray diffraction [31,32]. Still, it was not possible to determine the evolution of the α’ phase with temperature, so the onset of the solid solution was not detected by diffraction. 

### 4.3. Effect of Strontium

As has been mentioned, the Si phase is observed both as a primary crystal and as an Al-Si eutectic. It can be seen in the literature that, in comparison with Al–Si alloys, the nucleation of primary silicon crystals in Zn–Al–Si alloys is promoted by the presence of zinc. Nevertheless, the addition of strontium promotes the generation of eutectic silicon. Thus, in our microstructure we find both. The presence of strontium in the alloy is expected to modify both primary and eutectic silicon [42,43]. Since strontium is present in all samples in equal proportions, the modification effect could not be evaluated.

However, the test results showed that when heat treating the material between 30 and 280 °C, the strontium ceased to be just a modifier and interacted with zinc to generate SrZn_13_. This is a factor that must be considered since, on the one hand, it can affect the final properties of the alloy, reducing the availability of zinc for the Mg–Zn phases; on the other hand, it can influence subsequent transformation processes that take place in its stability range. Given the interactions that occur, it should be considered whether an alternative modifier, such as sodium or rare earth elements [44], should be used. 

## 5. Conclusions

The design and manufacturing of multicomponent light alloys give rise to non-conventional microstructures. In order to determine their potential applications, it is necessary to analyze their evolution with temperature. In this work, the multicomponent Al_58_Zn_28_Si_8_Mg_6_ alloy was studied with CALPHAD methods and then cast and heat-treated under several conditions. Characterization was carried out by X-ray thermo-diffraction, differential scanning calorimetry, and electron microscopy. 

As a result, a total of eight phases were identified in the alloy in the 30–380 °C temperature range: α-Al, α’-Al, Zn, Si, Mg_2_Si, MgZn_2_, Mg_2_Zn_11_, and SrZn_13_. The microstructures obtained at room temperature were metastable and the precipitation of Zn from the α’ phase occurred over the course of months. 

Moreover, the thermal transitions below 360 °C could be determined; that is, the dissolution and precipitation of Zn and dissolution of Mg_2_Zn_11_. Since the MgZn_2_ and Mg_2_Si phases dissolved above 360 °C, where partial melting may occur, those precipitates are not expected to harden the matrix, as they do in conventional 3xx.x and 7xx.x aluminum alloys with solution and precipitation treatments. However, two remarks should be made. First, a room temperature X-ray diffraction test performed immediately after the Q380 quenching treatment could offer valuable information about the solid solution capability of the alloy and enable a more direct comparison with the simulation results. Second, it was observed that the proportion of MgZn_2_ and Mg_2_Zn_11_ phases was highly dependent on the thermal history, so the microstructure of the alloy is still susceptible to adaptation by heat treatment.

It should be noted that strontium was added to modify silicon phases. Although it was a minor element, as well as Fe and Cu, it interacted with zinc between 30 and 280 °C. This fact should be considered during the postprocessing (thermal and/or thermomechanical treatments) in that temperature range, as it could have unexpected effects. 

Finally, it is clear from the experimental results that the used database is not designed to account for high percentages of alloying elements and thus is not able to accurately predict the actual phase evolution in the material; the Al–Zn system turned out to be tricky due to the metastable α’ phase, and the precipitation of MgZn_2_ in equilibrium conditions was predicted at a temperature that was too low. Nevertheless, the general guidelines given by CALPHAD methods, combined with SEM, DSC, and X-ray thermo-diffraction results, were able to give us a satisfactory understanding of the alloy’s behavior at different temperatures.

## Figures and Tables

**Table 1:** Chemical composition of the alloy analyzed by ICP.

	Al	Zn	Mg	Si	Cu	Fe	Sr
wt. %	41.15	48.40	4.11	5.83	0.43	0.05	0.03
at. %	57.56	27.93	6.38	7.83	0.26	0.03	0.01

**Table 2:** Heat treatments for each sample condition. The two-step solution treatment in Q360 and Q380 was applied to prevent any partial melting during one-step solution treatment at the solution temperature.

Sample Condition	Heat Treatment
as-cast	None.
Eq280	Heated to 280 °C and immediately slowly cooled (10 °C/min) to room temperature.
Eq360	Heated to 360 °C and immediately slowly cooled (10 °C/min) to room temperature.
Q280	Solution treated for 24 h at 280 °C, then water quenched to room temperature.
Q310	Solution treated for 24 h at 310 °C, then water quenched to room temperature.
Q360	Solution treated for 24 h at 325 °C, then heated and kept at 360 °C for 24 h and water quenched.
Q380	Solution treated for 24 h at 325 °C, then heated and kept at 380 °C for 24 h and water quenched.

**Table 3:** Experimental and theoretical temperatures for the reactions observed in the X-ray thermo-diffraction tests.

Reaction	Experimental T (°C) in Al_58_Zn_28_Si_8_Mg_6_ Alloy	T (°C) in Al–Mg–Zn System [40]
(Al) + (Zn) → (Al, Zn)	~285	-
(Al) + (Zn), Mg_2_Zn_11_ → (Al, Zn)	-	277
Mg_2_Zn_11_ + (Al) → MgZn_2_ + (Al, Zn)	~350	331
